# PACAP38 in human models of primary headaches

**DOI:** 10.1186/s10194-017-0821-3

**Published:** 2017-11-23

**Authors:** Håkan Ashina, Song Guo, Anne L. H. Vollesen, Messoud Ashina

**Affiliations:** Department of Neurology, Danish Headache Center, Rigshospitalet Glostrup, Faculty of Health and Medical Sciences, University of Copenhagen, Copenhagen, Denmark

**Keywords:** PACAP38, Human provocation models, Primary headaches, PAC_1_, Migraine

## Abstract

**Background:**

To review the role of PACAP38 in human models of primary headaches, discuss possible mechanisms of PACAP38-induced migraine, and outline future directions.

**Discussion:**

Experimental studies have established PACAP38 as a potent pharmacological “trigger” molecule of migraine-like attacks. These studies have also revealed a heterogeneous PACAP38 migraine response in migraine without aura patients. In addition, findings from brain imaging studies have demonstrated neuronal and vascular changes in migraine patients both ictally and interictally after PACAP38 infusion.

**Conclusion:**

Human migraine models have shed light on the importance of PACAP38 in the pathophysiology of primary headaches. These studies have also pointed to the PAC_1_ receptor and the PACAP38 molecule itself as target sites for drug testing. Future research should seek to understand the mechanisms underlying PACAP38-induced migraine. The results from an ongoing proof of concept randomized clinical trial may reveal the therapeutic potential of anti-PAC_1_ receptor antibodies for migraine prevention.

## Background

Much research effort has been devoted to studying the pathophysiology of primary headaches using human experimental models, which have led to the discovery of novel headache-eliciting signaling pathways and new drug targets [1]. In this context, pituitary adenylate cyclase-activating polypeptide (PACAP) has over the past decade emerged as a key signaling molecule implicated in migraine [2] and possibly also in cluster headache [3].

PACAP belongs to the glucagon/secretin superfamily of peptides together with vasoactive intestinal polypeptide (VIP) [4] and exists in two bioactive forms: a 38 amino acid form (PACAP38) and a truncated 27 amino acid form (PACAP27) [5]. PACAP38 is present in first-order neurons in the trigeminal ganglion [6], second-order neurons in the trigeminal nucleus caudalis (TNC) [7], and dorsal horn of the human spinal cord [8]. In addition, PACAP38 has also been identified in the otic and sphenopalatine ganglia [9], as well as in the cerebral cortex, cerebellum, brain stem and hypothalamus [10].

The effect of PACAP38 is mediated through three G-protein coupled receptors (PAC_1_, VPAC_1–2_) [11], two of which (VPAC_1–2_) hold equal affinity for PACAP38 and VIP, while the PAC_1_ receptor has a much higher affinity for PACAP38 [12]. The distribution of all three receptors has been documented in trigeminal, otic and superior cervical ganglia [13], as well as in cerebral and meningeal arteries [14]. Upon activation, all receptors cause downstream production of cyclic adenosine monophosphate (cAMP) through adenylate cyclase (AC) stimulation [15]. Studies have reported that the VPAC_1–2_ receptors play a role in vasodilation and mast cell degranulation [16–20], whereas one study in rats implicated the PAC_1_ receptor in pro-nociceptive transmission [21].

The headache-inducing effect of PACAP38 has been extensively studied in both healthy volunteers and migraine without aura (MO) patients. This has sparked an interest in pursuing specific treatment options targeting the PACAP38 molecule [22] or its PAC_1_ receptor [23]. Future randomized clinical trials (RCTs) will fully uncover whether PACAP38 or PAC_1_ receptor blockade could be a promising new approach in treating primary headaches.

In this review, we focus on human headache models using PACAP38 as a pharmacological “trigger” of migraine-like attacks. We then consider methodological aspects and limitations. Finally, we outline future perspectives and the therapeutic potential of anti-PACAP38 treatment to address unmet patient needs.

## PACAP38 migraine models

Birk et al. [24] for the first time systematically investigated PACAP38-induced headache and cerebral hemodynamics in 12 healthy volunteers. In this and the following described studies, healthy volunteers were identified as subjects who had no prior history of migraine and no first-degree relatives suffering from migraine. Ten out of 12 participants (83%) reported mild to moderate headache following PACAP38 infusion over 20 min, while no effect was observed on regional cerebral blood flow. There was a minor dilation of the middle cerebral artery (MCA) recorded by a transcranial Doppler (TCD) after PACAP38 infusion. However, certain limitations of the TCD method should be acknowledged. The TCD method assesses MCA velocity, which is dependent on the blood flow and the cross-sectional area of the artery. To interpret reduced velocity as arterial dilation, it requires that cerebral blood flow is constant despite heart rate variability and different angles of insonation. A more detailed description of methodological considerations on arterial measurements by TCD has recently been reviewed [25]. In healthy volunteers, the dose-response to 5, 10, 15, and 20 pmol kg^−1^ min^−1^ was investigated in three participants [24]. In all three cases the infusion was aborted after 10 pmol kg^−1^ min^−1^ due to 40–50% increases in heart rate – probably compensatory to the vasodilating effect of PACAP38. Following these observations, which have recently been confirmed in a dose-response study [26]; a dose of 10 pmol kg^−1^ min^−1^ is considered the optimal dose for experimental provocation studies.

Given that experimentally provoked attacks are not spontaneous according to the International Headache Society (IHS) criteria [27], different criteria for experimentally induced migraine attacks have been introduced [28]. The provoked migraine attacks should either fulfill IHS criteria C and D for MO [25] or mimic the usual migraine attack experienced by the patient and subsequent response to treatment with acute rescue medication [28]. To investigate the migraine-inducing effects of intravenous PACAP38 infusion, Schytz et al. [29] performed a double-blind, placebo-controlled crossover studies in 12 healthy volunteers and 12 MO patients. The authors hypothesized that PACAP38 infusion would induce headache in controls and migraine-like attacks in MO patients. All controls reported headache after PACAP38 infusion, while two controls also experienced migraine-like attacks. In MO patients, 7 out of 12 subjects (58%) reported migraine-like attacks after PACAP38 infusion compared with zero after placebo. Interestingly, the median time to peak headache score (4 h, range 0–12 h) in MO patients following PACAP38 provocation was similar to those reported in calcitonin gene-related peptide (CGRP) (5 h, range 2-9 h) and glyceryl trinitrate (GTN) (5.5 h, range 3–10 h) provocation studies [30, 31]. Moreover, the authors assessed the vascular effects of PACAP38 infusion on the MCA by TCD and the superficial temporal artery (STA) by dermascan ultrasonography during the hospital phase of the study (0-2 h post infusion) [29]. In MO patients, PACAP38 infusion caused a modest MCA dilation of 9.5% compared to baseline, while a more marked dilation of 37.5% was found in the STA. This study yielded two important findings. First, PACAP38 induced migraine-like attacks in 58% of MO patients, while no attacks were reported after placebo. Secondly, prolonged cranial artery dilation suggested a possible role of vascular mechanisms in PACAP38-induced migraine.

Magnetic resonance angiography (MRA) constitutes a superior method for measuring vessel diameter compared to TCD and provides more precise measurements of circumferential arterial changes [32]. All of the described provocation studies using TCD and MRA only assessed vascular effects in the middle meningeal artery (MMA), STA, and MCA [24, 29, 33, 34]. Using MRA, a double-blind, placebo-controlled study investigated the effect of PACAP38 infusion on the MCA and MMA in healthy volunteers [33]. The MMA was selected because it is the main artery supplying the dura mater and one previous study demonstrated MMA (but not MCA) involvement in CGRP-induced headache in healthy volunteers [35]. The major finding of the MRA study [33] was that PACAP infusion caused a long-lasting dilation (> 5 h) of the MMA co-occurring with headache, while no effect was found on the MCA circumference. In addition, subcutaneous injection of sumatriptan reversed the MMA dilation and headache, whereas the MCA circumference was unaltered. It is possible that PACAP38 does not reach its receptors on the smooth muscle cells in the MCA. In support, in vitro studies [36] reported a vasodilatory effect of PACAP38 on the rat and human MCA when applied abluminally but *not* luminally. The question is whether MMA dilation with co-occurring headache following PACAP38 infusion and the subsequent MMA constriction with co-occurring headache relief following sumatriptan reflect the importance of the MMA in migraine generation and cessation. It should be noted that sumatriptan is a 5-HT1B/1D receptor agonist that was originally developed as vasoconstrictor acting through receptor binding on cranial vessels [37]. However, its exact mechanism of action in relation to migraine remains a highly debated topic [38]. In healthy volunteers, subcutaneous injection of sumatriptan caused constriction of the STA, MMA, and MCA [39]. However, the same authors found a significantly smaller intracerebral arterial constriction compared with the constriction of extracerebral arteries – suggesting a primarily peripheral site of action for triptans. In the context of human provocation studies, subcutaneous injection of sumatriptan caused co-occurring MMA constriction and amelioration of migraine-like attacks following both PACAP38 [33] and CGRP [40] infusion. In both provocation studies [33, 40], no sumatriptan effect was found on the MCA circumference.

An interesting aspect to consider is that even though VIP belongs to the same family of peptides as PACAP38 [41], it does not induce migraine attacks in MO patients [42]. VIP infusion only induced dilation of cranial arteries and mild headache [42]. To further examine this issue, one MRA study investigated the response to intravenous infusion of PACAP38 or VIP in MO patients [34]. Sixteen out of 22 patients (73%) reported delayed migraine-like attacks following PACAP38 infusion, whereas only 4 out of 22 (18%) did so after VIP infusion. Moreover, this study found that both PACAP38 and VIP induced STA and MMA dilation, while the MCA remained unaffected. The PACAP38-induced vasodilation was longer-lasting (> 2 h) than the VIP-induced vasodilation which normalized after 2 h. Interestingly, there was no difference in arterial circumference between the pain and non-pain side during PACAP38-induced migraine-like attacks in 9 patients. Subcutaneous injection of sumatriptan reduced headache intensity and caused constriction of only the extracranial arteries. Another key finding from this study was that plasma levels of PACAP38 were elevated in MO patients who developed migraine-like attacks compared to those who did not 60 min after PACAP38 infusion. Since plasma PACAP38 has a half-life of 3.5 min [24], a complete clearance of exogenous PACAP38 is expected 60 min after the start of infusion. To explain this, the authors suggested three possible mechanisms [34]: 1) impaired elimination; 2) endogenous release; 3) de novo synthesis. However, when the data from this study [40] was later pooled with data from a second study from the same research group [43] to increase power and sample size, there was no difference in pre-ictal PACAP38 plasma levels between patients who developed migraine-like attacks (*n* = 39) compared to those who did not (*n* = 15). To our knowledge, no study has investigated the underlying mechanisms of PACAP38-induced prolonged vasodilation.

A resting-state functional magnetic resonance imaging (fMRI) study examined the involvement of specific changes in cerebral network connectivity before and during PACAP38-induced migraine-like attacks in MO patients [44]. VIP was used as active placebo. Resting state fMRI is a method to evaluate regional interactions in cerebral connectivity when a subject is not performing an explicit task. Patients were scanned 30 min, 130 min, and 310 min after PACAP38-infusion, unless they reported migraine-like attacks. In the event of migraine-like attacks, immediate scans were performed. The study found abnormal cerebral connectivity in all the investigated cerebral networks (salience, sensorimotor, and default mode) at the onset of migraine-like attacks after PACAP38 infusion when compared to outside of the attacks [44]. No alterations in cerebral connectivity were found following VIP infusion. These findings are interesting because those three networks have been implicated in the processing of nociceptive and emotional signals [45–48]. To solidify the importance of these findings, the authors suggested that a similar experiment should be conducted before and at the early phase of spontaneous migraine attacks.

One provocation study has also examined the incidence of premonitory symptoms induced by intravenous PACAP38 administration in MO patients [49]. Premonitory symptoms occur hours to 2 days prior to the migraine attack [28] and most commonly present themselves as unusual fatigue, neck stiffness, and poor concentration. It has previously been reported that 36% of migraine patients experience premonitory symptoms following GTN infusion [50]. Following PACAP38-infusion [49], 72% and 48% of the patients experienced migraine-like attacks and premonitory symptoms, respectively. Interestingly, CGRP did not induce premonitory symptoms in the same group of patients. In addition, there was no difference of premonitory symptoms in patients who developed attacks versus those who did not. These findings are interesting because premonitory symptoms are considered a marker of CNS involvement. However, the study did not include a healthy control group or placebo-treated patients. Therefore, we cannot exclude that the observed association between PACAP38 infusion and premonitory symptoms could be due to substance-related side effects.

As we reflect on the headache-inducing capabilities of PACAP38, it is interesting that some MO patients develop migraine-like attacks while others do not. The question is whether fluctuating susceptibility could be due to genetic variations among migraine patients. Genetic studies have documented that genetic enrichment of certain risk factor genes constitute a predisposition to developing migraine [51–53]. To address this issue, one study [54] stratified patients into two groups: one group with high family load (≥ 2 first-degree relatives with MO) and one group with low family load (≤ 1 first-degree relatives with MO). In addition, genotyped patients were stratified based on risk allele status. This study revealed no association of hypersensitivity to migraine following PACAP38 administration based on family load and migraine-associated risk allele status in 32 genotyped MO patients.

## Possible mechanisms of PACAP38-induced migraine

Several possible mechanisms on the migraine-inducing effect of PACAP38 have been suggested: vasodilatation via cAMP, mast cell degranulation, parasympathetic involvement, activation of sensory afferents by the cAMP-signaling pathway or via the PAC_1_ receptor, and central effects.

### Vasodilation via cAMP

PACAP38 is a powerful dilator of cerebral arteries [29, 33] and its effect is mediated through a cAMP-dependent signaling pathway [15]. In relation to migraine, one human experimental study provided evidence for cAMP upregulation in migraine induction in MO patients after cilostazol (phosphodiesterase 5 inhibitor) administration [28]. Interestingly, cilostazol is known to produce a long-lasting dilation of cerebral arteries [55] and PACAP38 induces long-lasting MMA-dilation (> 2 h) [34]. To what extent the long-lasting MMA-dilation contributes to PACAP38-induced migraine remains unknown.

### Mast cell degranulation

Another interesting aspect to consider is the role of mast cell degranulation in PACAP38-induced migraine. Mast cells are found throughout the human organism and play an important role in the immediate response to hypersensitivity reactions [56]. Upon activation, mast cells release soluble mediators (e.g. histamine, TNF-α, and tryptase) into the circulation. Interestingly, histamine induces migraine-like attacks in 70% of MO patients [57]. Furthermore, mepyramine (a histamine H_1_ receptor blocker) pretreatment abolished both immediate and delayed histamine-induced migraine-like attacks in the same group of MO patients [57]. In relation to PACAP38, one in vitro study found that PACAP38 induced mast cell degranulation in dural and peritoneal mast cells in rats [58]. Furthermore, PACAP38-induced MMA dilation was abolished in both mast cell depleted and antihistamine pretreated rats [59]. Therefore, the authors suggested that mast cell mediated histamine release was implicated in PACAP38-induced MMA-dilation [59]. In rats, mast cell degranulation activates and sensitizes meningeal dural afferents [60]. Interestingly, PACAP38 has a more potent degranulatory effect on dural mast cells in rats compared with PACAP27 and VIP [58]. Thus, it seems conceivable that the effect of PACAP38 on mast cell degranulation is primarily mediated through the PAC_1_ receptor because VIP had a lesser effect on mast cells. However, the same study [58] also found no effect of PAC_1_ receptor agonist (maxadilan) on mast cells, while PAC_1_ receptor antagonism mediated mast cell degranulation. Thus, it could be speculated whether PACAP38 elicits its effect on mast cells through a distinct target from the PAC_1_ receptor. In humans, PACAP38-associated flushing and heat-sensation terminated following anti-histamine treatment [29]. It should, however, be noted that two human provocation studies collected peripheral plasma levels of inflammatory mast cell mediators (tumor necrosis factor alpha and tryptase) in MO patients after PACAP38 infusion [34, 43]. These studies found no changes in plasma tumor necrosis factor alpha and tryptase. Whether peripheral plasma changes reliably reflect cranial release of mast cell mediators remains unknown. Also, timing of collection might play a role in detecting altered peripheral plasma levels of mast cell mediators. Thus, mast cell degranulation cannot be completely dismissed as a mediator in the migraine-inducing mechanisms of PACAP38.

### PACAP38 in the parasympathetic system

PACAP38 has been identified in both the sensory [6, 7] and parasympathetic system [61]. The parasympathetic distribution of PACAP38 stems from the sphenopalatine and otic ganglia [9], as well as from parasympathetic perivascular nerve fibers [62]. It has been suggested that parasympathetic efferent fibers play a role in the trigeminovascular system by releasing neuropeptides, such as PACAP38, involved in nociceptive transmission [63]. Interestingly, VIP is also present in both the sphenopalatine and otic ganglia [64], but *no* VIP immunoreactivity has been found in the trigeminal ganglion [65]. These data indicate that PACAP38 has two sites of origin unlike VIP: the parasympathetic system and the sensory system. In this context, it is also interesting that the PACAP and VIP molecules are parasympathetic biomarkers and both are less prominently expressed in the dura mater and trigeminal ganglion compared with CGRP, while being more prominently expressed in cerebral vessels [66]. Therefore, the authors speculated that PACAP has a larger parasympathetic distribution and a minor sensory distribution. These data suggest PACAP38 might function primarily as a neuropeptide in parasympathetic pathways underlying migraine, while CGRP acts as a neuropeptide in sensory pathways underlying migraine. However, to what extent parasympathetic efferent fibers play a role in PACAP38-induced migraine remains a subject for further investigation.

### Activation of sensory afferents by the cAMP-signaling pathway or via the PAC_1_ receptor

In the sensory nervous system, PACAP38 is present in first-order neurons in the trigeminal ganglion [6] and in second-order neurons in the TNC [7]. All three PACAP38 receptors upregulate cAMP [14] and PACAP38 receptors have been detected in both the trigeminal ganglion [13] and TNC [67]. In view of findings from human experimental data, migraine-like attacks after PACAP38 infusion could be explained by modulation of dural or extracranial trigeminal nociceptors outside of the BBB [68]. The presence of all PACAP38 receptors has been identified in the vessel wall of human cerebral arteries [13]. Hence, it could be speculated that PACAP38 upregulates intracellular cAMP in trigeminal nociceptors following PAC_1_ receptor activation. This mechanism could possibly initiate a neurobiological cascade resulting in migraine attack development. Indeed, CGRP and cilostazol also upregulate intracellular cAMP [69, 70] and both are potent triggers of migraine-like attacks in MO patients [28, 71]. In this context, it is interesting that elevated intracellular cAMP levels have been associated with activation of trigeminal neurons [72] and meningeal nociceptors [73]. Given that both PACAP38 and CGRP act on cell membrane receptors one would expect a similar median time to migraine onset after infusion. However, direct comparison of different groups of patients is problematic. To directly compare PACAP38 and CGRP migraine responses would require head to head comparison in the same group of patients. To date, this has not been investigated. One ongoing RCT is currently investigating the ability of CGRP blockade to prevent PACAP38-induced migraine [74].

### PACAP38 and central effects

It has been reported that PACAP38 is able to cross the BBB by a saturable transport mechanism [75] and that 0.053% of PACAP38 passes the BBB after 5 min following intravenous infusion [76]. Animal models have implicated PACAP38 in central nociceptive transmission [77] and in rats intrathecal capsaicin elevated PACAP levels in the cerebrospinal fluid [78]. Hence, spinal cord C-fibers might release PACAP upon activation. Therefore, PACAP38 might modulate nociceptive input through its PAC_1_ receptor which is expressed on second-order trigeminal neurons [7]. Furthermore, the hypothalamus contains the most abundant population of PACAP38-containing neurons [79] and its activation has previously been associated with premonitory symptoms in GTN-provoked migraine attacks [80]. Interestingly, MO patients reported premonitory symptoms after PACAP38 infusion [49]. Yet, we do not have sufficient data to confirm or refute a central effect of PACAP38-induced migraine-like attacks.

## Discussion and future perspectives

The human experimental studies have demonstrated the potency of PACAP38 as a pharmacological “trigger” of migraine-like attacks [29, 34, 44, 49, 54]. Yet, there are several methodological limitations and aspects worth pointing out that should be optimized in future study designs. In the following, we will discuss: 1) Plasma PACAP38 as a biochemical marker in human experimental models; 2) heterogeneity of the PACAP38 response in MO patients; 3) MRA biomarkers of PACAP38-induced migraine-like attacks; 4) future experimental models using PACAP27.

### PACAP38 as a biochemical marker

In migraine patients, PACAP38 plasma levels have been measured during both spontaneous [81, 82] and PACAP38-induced [43] migraine attacks. Tuka et al. [81] reported elevated ictal PACAP38 plasma levels during spontaneous migraine attacks relative to interictal PACAP38 plasma levels. Migraine sufferers also had lower interictal PACAP38 plasma levels compared with healthy controls. Another study reported elevated PACAP38 plasma levels during migraine attacks and found that subsequent sumatriptan administration was associated with a decrease in PACAP38 plasma levels [82]. In contrast, pooled data analysis from two PACAP38 provocation studies found no pre-ictal phase increase in PACAP38 plasma levels in MO patients who experienced migraine-like attacks [37]. The conflicting data could be explained by assay variation [82–84] and differences in timing of measurements. It is also debatable whether peripheral plasma measurements reliably reflect cranial PACAP38 release and therefore the jugular vein might constitute a more precise site of collecting blood samples. It should be noted that one study reported no difference in extracranial and cranial CGRP plasma levels in healthy volunteers [85]. In addition, two of the studies [81, 82] included both MO patients and migraine with aura (MA) patients. Thus, a different PACAP-response cannot be excluded in MO patients compared with MA patients. For future human provocation studies, it would be interesting to measure PACAP38 plasma levels after sumatriptan administration when using PACAP38 or other pharmacological “triggers” such as CGRP and cilostazol. Here, it would be important to have a placebo-controlled design; otherwise, PACAP38 could possibly decrease spontaneously over the course of a migraine attack. The exact antimigraine mechanism of sumatriptan remains unknown; thus, it could be speculated that a reduction in PACAP38 plasma levels might play a role.

### Heterogeneity of the PACAP38 response in MO patients

**Fig. 1:**
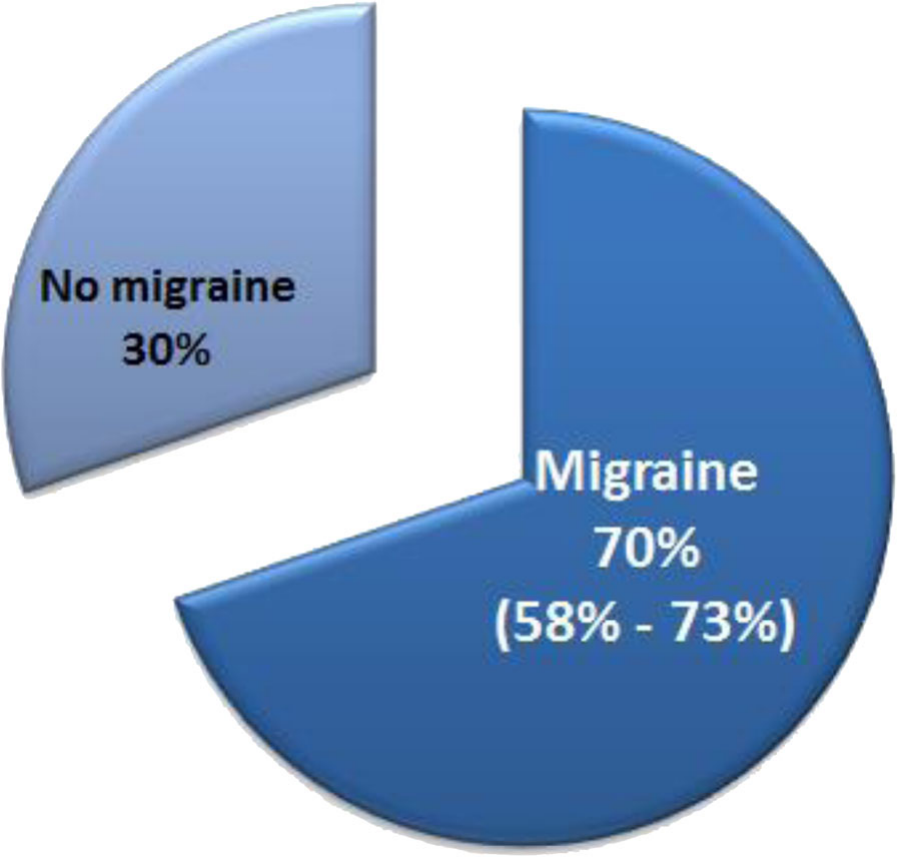
Proportion (median and range) of patients who developed migraine-like attacks and of patients who did not develop migraine-like attacks after PACAP38 infusion [29, 34, 54]

Human experimental studies have revealed a heterogeneous PACAP38 response in MO patients in that some develop migraine-like attacks while others do not (Table 1). In a total of three PACAP38 provocation studies, 46 out of 66 (70%) MO patients experienced migraine-like attacks (Fig. 1). Giving this evidence, the question is whether susceptibility to migraine follows a fluctuating pattern. Indeed, two studies suggested that the likelihood of migraine attack development exhibits innate variations [86, 87] – suggesting a migraine threshold that varies over time. Thus, it raises the issue of whether PACAP38-induced migraine-like attacks can only be induced at certain points in a migraine susceptibility cycle. This seems questionable because a small PACAP38 dose-response pilot study found that PACAP-induced migraine-like attacks seem to be reproducible in MO patients [26]. Furthermore, the large sample size of MO patients who developed migraine-like attacks after PACAP38 infusion (46 out of 66) deem it improbable that all 46 patients were in a migraine susceptible phase. To dissect this issue, MO patients with few attacks on a yearly basis should undergo PACAP38 provocation to clarify the importance of possible cyclic migraine induction variability. In relation to this, one provocation study [88] found no relation between headache frequencies in MO patients and GTN-induced headache.

**Table 1:** Overview of PACAP38 provocation studies in migraine without aura (MO) patients

Reference	Study design	Number of MO patients who developed migraine-like attacks	Total number of MO patients	Major findings
Schytz et al. [29]	Double-blind crossover	7	12	PACAP38 induces migraine-like attacks
Amin et al. [34]	Double-blind randomized	16	22	PACAP38-induced migraine is associated with sustained dilation of extracranial arteries
Amin et al. [44]	Double-blind crossover	18	24	PACAP38-induced migraine-like attacks is associated with altered functional cerebral connectivity
Guo et al. [49]	Double-blinded	23	32	PACAP38 induces premonitory symptoms
Guo et al. [54]	Double-blinded	23	32	PACAP38 response is not associated with high family load or risk allele status

Future provocation reproducibility studies should also include a three-arm crossover design with subjects randomized to PACAP38; PACAP38; Placebo. This would permit blinded assessment of PACAP38 reproducibility while controlling for placebo response. Following this, phenotyping migraine sufferers in PACAP38 responders versus non-responders could be used to possibly predict efficacy of drugs targeting the PACAP38 molecule or its PAC_1_ receptor. This would delineate PACAP38 as a reliable biomarker of the migraine. Future studies should also investigate whether blockade of PACAP38 or its PAC_1_ receptor may prevent the migraine-inducing effects of PACAP38. Moreover, studies in MA and FHM patients are needed to cover the whole migraine spectrum with respect to PACAP38 provocation. The same need exists for PACAP38 provocation studies in cluster headache and tension-type headache patients.

### Magnetic resonance imaging

Advanced brain imaging in human experimental models provides a unique opportunity to identify biomarkers specific to primary headaches. PACAP38 provocation studies have demonstrated neuronal and vascular changes taking place in the brains of migraine sufferers both ictally and interictally. For this reason, future studies should investigate whether PACAP38 or PAC_1_ receptor blockade is able to prevent PACAP38-induced MMA dilation. If this proves to be the case, the question is whether blockage of MMA dilation co-occurs with blockage of PACAP38-induced migraine-like attacks. This would in particular be interesting in a population of migraine patients that have previously been stratified as PACAP38 responders.

### PACAP27 as pharmacological “trigger” of primary headaches

The migraine-inducing effects of PACAP38 have been well-documented but to date no study has investigated the migraine-inducing effect of PACAP27. It is the less abundant bioactive form of the PACAP molecule, but displays similar affinity to the PAC_1_ receptor as PACAP38 [89]. Interestingly, the role of the 28-to-38 segment of the PACAP molecule seems to be important for two reasons. First, one study [90] indicated that the 28-to-38 segment could possibly play a role for the BBB transporter to recognize and transport the PACAP molecule across the BBB. Secondly, PACAP38 has a half-life of less than 5 min in human plasma in vitro, while PACAP27 displays a relative lack of degradation [91]. For these reasons, it would be relevant to investigate the response to PACAP27 provocation in migraine and cluster headache patients.

## Conclusion

Great advances have been made over the past decade in understanding the pathophysiology of migraine using PACAP38 as a pharmacological “trigger”. Knowledge acquired from these human experimental studies has shed light on the PACAP38 molecule or its PAC_1_ receptor as potential therapeutic drug targets. Nonetheless, the PACAP38 migraine-specific mechanisms of action have not been fully clarified and its involvement in cluster headache and tension-type headache remains a subject for investigation. Future studies will seek to refine design and execution; thereby, paving the way to delineate biomarkers of primary headache disorders.

## Abbreviations

AC: Adenylate cyclase
cAMP: Cyclic adenosine monophosphate
CGRP: Calcitonin gene-related peptide
fMRI: Functional magnetic resonance imaging
GTN: Glyceryl trinitrate
IHS: International Headache Society
MA: Migraine with aura
MCA: Middle cerebral artery
MMA: Middle meningeal artery
MO: Migraine without aura
MRA: Magnetic resonance angiography
PACAP: Pituitary adenylate cyclase-activating polypeptide
RCTs: Randomized clinical trials
STA: Superficial temporal artery
TCD: Transcranial Doppler
TNC: Trigeminal nucleus caudalis
VIP: Vasoactive intestinal polypeptide
